# Comparative Analysis of Methods for Identifying Recurrent Copy Number Alterations in Cancer

**DOI:** 10.1371/journal.pone.0052516

**Published:** 2012-12-20

**Authors:** Xiguo Yuan, Junying Zhang, Shengli Zhang, Guoqiang Yu, Yue Wang

**Affiliations:** 1 School of Computer Science and Technology, Xidian University, Xi'an, P. R. China; 2 Bradley Department of Electrical and Computer Engineering, Virginia Polytechnic Institute and State University, Arlington, Virginia, United States of America; 3 Department of Mathematics, Xidian University, Xi'an, P. R. China; Tel Aviv University, Israel

## Abstract

Recurrent copy number alterations (CNAs) play an important role in cancer genesis. While a number of computational methods have been proposed for identifying such CNAs, their relative merits remain largely unknown in practice since very few efforts have been focused on comparative analysis of the methods. To facilitate studies of recurrent CNA identification in cancer genome, it is imperative to conduct a comprehensive comparison of performance and limitations among existing methods. In this paper, six representative methods proposed in the latest six years are compared. These include one-stage and two-stage approaches, working with raw intensity ratio data and discretized data respectively. They are based on various techniques such as kernel regression, correlation matrix diagonal segmentation, semi-parametric permutation and cyclic permutation schemes. We explore multiple criteria including type I error rate, detection power, Receiver Operating Characteristics (ROC) curve and the area under curve (AUC), and computational complexity, to evaluate performance of the methods under multiple simulation scenarios. We also characterize their abilities on applications to two real datasets obtained from cancers with lung adenocarcinoma and glioblastoma. This comparison study reveals general characteristics of the existing methods for identifying recurrent CNAs, and further provides new insights into their strengths and weaknesses. It is believed helpful to accelerate the development of novel and improved methods.

## Introduction

Identifying recurrent copy number alterations (CNAs) in cancer genomes is an important step in locating cancer driver genes and understanding the mechanisms of tumor initiation. Many human cancers including ovarian serous carcinoma [1], lung adenocarcinoma [2], glioblastoma multiforme [3], and other types of cancers [4], [5], have been largely explored by analyzing CNAs. However, the identified CNAs with high frequency of occurrence across multiple samples only account for a small fraction of clinically or biologically relevant aberrations for many cancers. The most common reason for missing some well-known driver mutations is that almost all cancers are heterogeneous [6], indicating that many recurrent CNAs only appear in a subset of samples (i.e., samples within subtypes) and accordingly their frequencies are less-extreme across the whole samples. For this challenge, a number of statistical and computational methods with promising results have been reported. They are divided into one-stage [7], [8], [9], [10] and two-stage approaches [3], [4], [11], [12], [13]. Many of them were reviewed and discussed by Rueda and Diaz-Uriarte in their latest paper [14].

One outstanding phenomenon of copy number profiles is that a part of markers are changed in identical regions in multiple genomes and the remainder markers are changed in random places of the genomes. Thus, the frequency of CNA occurrence across samples is usually used to help distinguish recurrent events from random markers. However, due to the complicated structures of copy number data, the identification of less-extreme recurrent CNAs is an extremely difficult task. Below we profile a real copy number dataset to show the complexity of CNAs, and further use it as an example to illustrate why the less-extreme CNAs are difficult to detect.

**Figure 1:**
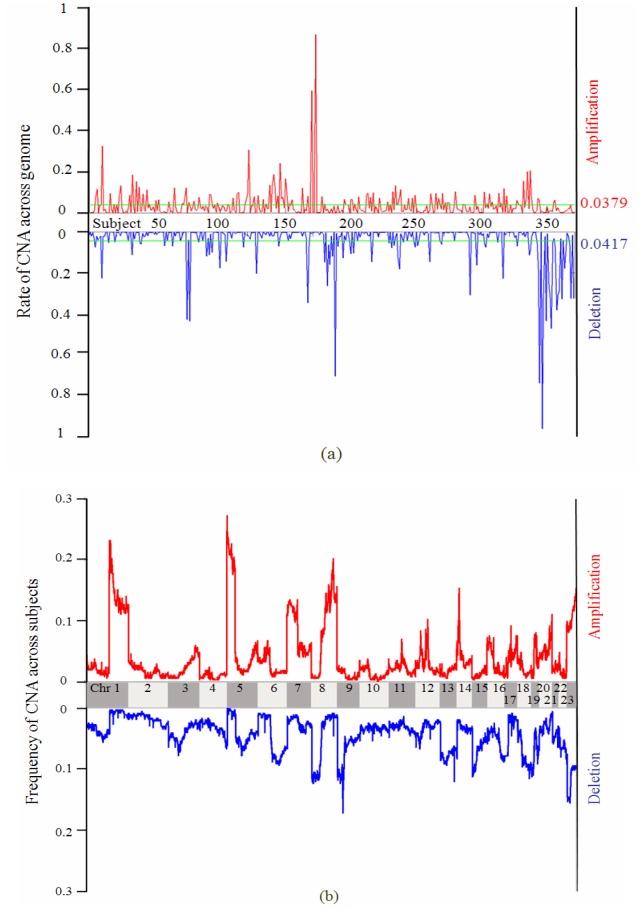
The rate of somatic CNA across the genome and the frequency of somatic CNA across the samples in a set of segmented lung cancer profiles. Here we use log_2_-ratios 0.322 (2.5 copies) and −0.415 (1.5 copies) to define amplifications and deletions. (a) The average rates of CNA for amplification and deletion among the 371 samples are 0.0379 and 0.0417, respectively. (b) A large part of amplifications and deletions are less than 0.1 in terms of frequency.

Figure 1a and Figure 1b depict the rate of CNA occurrence across the entire genome and its frequency across the samples in a set of lung cancers, which contains 371 samples and 216,327 markers [3], [5]. It can be noted from the figures that most of the markers are changed (amplified or deleted) in at least one sample and many of them are overlapped by a part of samples. Additionally, the sizes of CNA regions vary from chromosome to chromosome. For a given set of *N* cancer samples, assuming all the observed CNAs are randomly distributed across the genome in each sample, the expected probability (E(*P*)) of one CNA marker shared by at least *n* samples (corresponding to a percentage *f* of the whole samples) can be estimated using Equation (1), and consequently the expected number (E(*l*)) of such shared markers in the genome can be expressed by Equation (2).
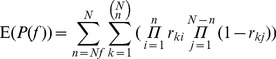
(1)

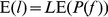
(2)where *L* is the length of the genome being analyzed; *r_ki_* and *r_kj_* are the CNA rates of the *i*-th and *j*-th samples in the *k*-th subset, which refers to the *k*-th combination of *n* samples chosen from the whole *N* samples. Here, the total number of combinations of choosing *n* from *N* is represented by 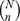.

**Figure 2:**
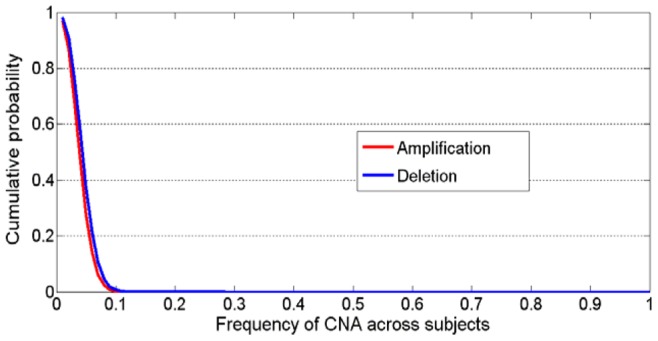
Cumulative probability as a function of the CNA frequency across samples for amplification and deletion in the sample set being considered.

Let us consider a set of 100 samples with each having 1000 markers, and in each sample the rates of CNA are 0.035 for amplification and 0.040 for deletion (these frequencies are relatively less than the means of the above lung cancer dataset). If we assume the CNAs are randomly placed in the genome, the probability of one marker shared by at least 100*f* (0< *f* ≤1) samples can be regarded as a cumulative probability, termed *P_c_*(*f* ) (shown in Equation (3)). For instance, *P_c_*(0.1) equals to 0.0027 in the case of amplification, indicating that the probability of one marker amplified in at least 10 (0.1 multiplies 100) samples is 0.0027. Figure 2 shows such cumulative probability versus the frequency of one CNA marker across the 100 samples. Consequently, the number of such markers in the whole genome can be estimated as 1000 *P_c_*(*f* ).
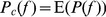
(3)


**Figure 3:**
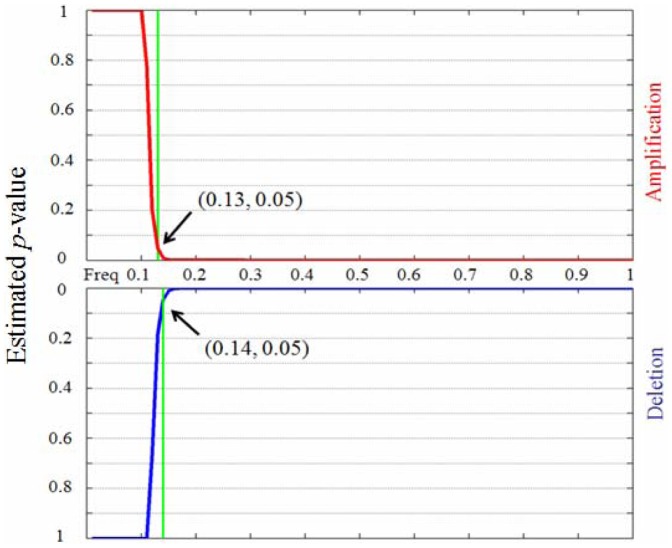
Estimated p-value as a function of CNA frequency in the sample set being considered. In the case of amplification, p-value (0.12) = 0.20 and p-value (0.13) = 0.05; in the case of deletion, p-value (0.13) = 0.18 and p-value (0.14) = 0.05.

If the frequency is used as a statistic to test the significance of CNAs individually, the estimated *p*-value for the marker with frequency *f* can be calculated using Equation (4), which is under the max-T procedure to control the family-wise error rate (FWER) [15]. For clearly understanding the relationship between the CNA frequency and its *p*-value, we demonstrate the *p*-value as a function of the frequency ranging from 0.01 to 1 for amplification and deletion separately in Figure 3. It can be noted that the *p*-value decreases with the increased frequency of the CNA, and particularly, *p*-value is 0.05 when *f* = 0.13 in the case of amplification and *p*-value equals to 0.05 when *f* = 0.14 in the case of deletion. These suggest that if a *p*-value cutoff 0.05 is employed, the CNA markers with frequency less than 0.13 for amplification (or less than 0.14 for deletion) could not be detected, while in real data such frequency may be of significant biological relevance since many CNAs may affect only a minority of cancer samples [3], [7].
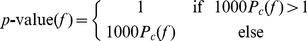
(4)


Generally, the frequency-statistic and random permutation of markers in the above example is just a basic strategy to test significance. To complement this strategy, many methods design various statistics and null distributions for this challenge. For example, STAC (Significance Testing for Aberrant Copy number) [4] proposes a new statistic “footprint” to score each marker and establishes the distribution under the null hypothesis that the observed CNA regions are equally placed anywhere across the genome; GISTIC (Genomic Identification of Significant Targets In Cancer) [3] scores each marker by combing frequency and amplitude, and constructs a semi-exactly approximated null distribution, and its extension GISTIC2.0 [11] considers the distinction of the background frequency between focal CNAs and broad CNAs and scores each marker proportional to its amplitude; CMDS (Correlation Matrix Diagonal Segmentation) [9] scores each marker based on its correlations with its surrounding sites and constructs a student’s *t* distribution; and DiNAMIC (Discovering Copy Number Aberrations Manifested In Cancer) [13] employs a summary statistic and a cyclic permutation scheme to generate the null distribution. In addition, to adjust statistic values and improve null distributions, many methods employ a peel-off algorithm to iteratively test CNAs [3], [13], [16], [17]. This will help much in identifying low-to-moderate-frequency (or/and low-to-moderate-amplitude) markers.

Along with recent advance of genomic technologies and rapid production of huge datasets, new methods with more sophisticated capabilities and features for detecting recurrent CNAs continue to emerge. However, the relative strengths and weaknesses of the existing methods are difficult to discern, due to the lack of comprehensive performance comparisons. This is a true problem especially from the perspective of biological researchers who need to choose a method for a dataset of interest. In this paper, we compare six classic and publicly available methods based on criteria including type I error rate, detection power, Receiver Operating Characteristics (ROC) curve and the area under curve (AUC), and computational complexity, so that users can quickly get an overview of them and their performance. Various simulation datasets and two real datasets obtained for lung adenocarcinoma and glioblastoma samples are used to evaluate the methods.

## Materials and Methods

### Methods for Identifying Recurrent CNAs

A variety of statistical and computational methods have been proposed recently for identifying recurrent CNAs. These methods can be categorized in different ways, such as frameworks, strategies for establishing null distributions, source codes, and so on. Generally, different cancer datasets have distinct profiles and patterns of copy number alterations, and they may require different computational methods for analysis, as there is no single method that could be suitable for all datasets. It is necessary to explore those methods that possess distinct features and different advantages. To mirror this, we carefully select six representative methods for assessment and comparison, based on their reported effectiveness in real applications. We list the six methods in Table 1 as well as their properties for an overview. These methods have been developed under different rationales in the latest six years and some of them have been widely used in cancer data analysis [2], [18], [19]. For a general understanding of them, we give a brief summary of their principles as follows.

**Table 1:** Methods evaluated in this comparison study.

Name	Framework	Statistic	Null distribution	Peel-off	Software
STAC	Two-stage	Frequency/Footprint	Permutation of segments onchromosome	No	JAVA, http://cbil.upenn.edu/STAC
GISTIC	Two-stage	G-score	Permutation of markers onwhole genome	Yes	Matlab, www.broad.mit.edu/cancer/pub/GISTIC
KC-SMART	One-stage	KSE (Kernel smoothed estimate)	Permutation of log2 value ineach marker	No	R package, http://bioc.ism.ac.jp/2.8/bioc/html/KCsmart.html
CMDS	One-stage	RCNA score	*t* distribution created basedon marker correlations	No	R package, https://dsgweb.wustl.edu/qunyuan/software/cmds
DiNAMIC	Two-stage	Summary statistic *T*	Cyclic permutation onchromosome or whole genome	Yes	R package, http://www.bios.unc.edu/research/genomic_software/DiNAMIC
GAIA	Two-stage	Aberration count	Permutation of markers onchromosome	Yes	R package, http://bioinformatics.biogem.it/download/gaia

For the “peel-off” column, ‘Yes’ and ‘No’ indicate whether the methods adopt peel-off procedure.

#### (1) STAC [4]

The input of STAC is a binary matrix *X*, in which each element *x_ij_* represents the status of *j*-th marker at sample *i*. Specifically, *x_ij_* = 1 stands for amplification (or deletion), *x_ij_* = 0 means normal. It analyzes amplification and deletion matrices separately, and tests significance of them in the same way. The null hypothesis behind STAC is that the observed CNA segments are randomly placed anywhere in the chromosome being considered [4], [17], hence permuted samples can preserve the original structures of the copy number data. STAC adopts two statistics, frequency of aberration and “footprint”, to assess *p*-values for each marker, and controls the family-wise error rate (FWER) based on the extreme right-hand tail probability [4], [13], [20].

The “frequency” for marker *x* is calculated as the proportion of samples sharing the aberration, while the “footprint” for marker *x* is calculated as a number of locations contained in a stack, which is a set of intervals containing *x* across samples [4]. The principle behind the “footprint” is that the tighter alignments of aberrations are less likely to be expected by chance and thus are more likely to suggest biologically relevant events, while the more relaxed alignments of aberrations might suggest passenger mutations with higher probability.

#### (2) GISTIC [3]

This method requires segmented input data with continuous log_2_-values resulted from single sample analysis methods such as CBS [21] and GLAD [22]. It permutes individual markers on the whole genome by assuming that the markers are independent [3], [17], and derives a semi-exact estimated null distribution based on the convolution function [3] of
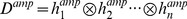
(5)where 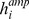
is the distribution (histogram) of amplification in the *i*-th sample. Based on the null distribution, GISTIC uses a *G*-score combining both frequency and amplitude (Equation 6) to assess the significance for each marker and corrects for multiple hypothesis testing through the Benjamini-Hochberg FDR procedure [23]. The same procedure is applied to the analysis of deletion and LOH (loss of heterozygosity).
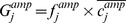
(6)where 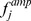
and 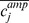 are the frequency of the amplification and the average amplitude of the *j*-th marker across the samples.

The intuition behind the *G*-score is that an aberration with higher amplitude and frequency is more likely to be a driver event. In order to relieve the side effect of peak regions with the highest amplitude and frequency, GISTIC adopts a “peel-off” algorithm to iteratively test the CNAs within the significant regions.

#### (3) KC-SMART [8]

Different from the above two methods, one-stage framework is embraced for this method without requiring a prior step of segmenting (smoothing) copy number profiles. The principle behind KC-SMART is that it imposes a kernel function at each location *m* to construct a statistic, kernel smoothed estimate (KSE) [8]:
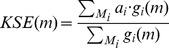
(7)where 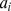 is a summed positive or negative log_2_-ratios across all samples for each location, 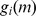 is a kernel function (e.g. flat-top Gaussian kernel function), and 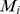 is a set of markers around location *m* and it is usually determined based on the width of the kernel function. Theoretically, this statistic considers the correlations among copy number data and incorporates information gained from neighboring markers.

To identify peak locations (i.e., recurrent CNAs), the method compares the observed KSE of each location against a null distribution which is established through permutations of individual log_2_-ratios on the genome being considered. To correct the effect of multiple hypotheses testing, KC-SMART adopts Bonferroni strategy by multiplying the assessed *p*-values using the total number of locations being tested.

#### (4) CMDS [9]

The input data to CMDS is largely similar to KC-SMART. This method doesn’t directly utilize the frequency and amplitude of copy number aberrations to construct test statistic. It assigns a RCNA score to each marker. The RCNA score is an averaged correlation value over the surrounding sites of the marker. The null hypothesis of CMDS is that there is no correlation between markers within chromosomes, thus it can be created by randomly permuting individual markers in the stretch of the chromosome being considered. To save computational time, CMDS uses the information from the observed correlation values in the copy number genome to establish a standard normal distribution, as a closely approximated *t* distribution. The multiple-testing effect is also corrected using Bonferroni strategy, exactly like the KC-SMART method.

The intuitive notion behind CMDS is that the copy number noise is not correlated while the recurrent CNAs are in high correlation. Another outstanding feature of CMDS is that it doesn’t analyze amplification and deletion separately, but uses the average copy number value over the predefined window across all samples and its significance level [9] to determine whether the corresponding marker is amplification or deletion. This is different from most other existing methods.

#### (5) DiNAMIC [13]

This method accepts both continuous raw signal and discrete segmented data. It adopts a global summary statistic that incorporates both frequency and amplitude of each marker for analyzing either amplification or deletion. Two novel features underlying DiNAMIC are concluded as follows. First, it employs a cyclic permutation strategy to generate the null distribution [13], [17], which preserves the structures of the original copy number data at a higher degree than most other methods such as STAC [4] and GISTIC2.0 [11]. Second, to increase the power for detecting less-extreme CNA markers, the method utilizes a “peel-off” algorithm different from that used by GISTIC [3], which assesses the significances of new regions by removing all aberrations overlapped by the previously detected recurrent regions, while DiNAMIC re-tests markers by generating a new null distribution on a new data matrix in which the previously detected markers *K* are null and the markers contribute to the significance of *K* are scaled using a factor.

This method is supposed to test one marker during each “peel-off” iteration procedure, thus computational cost will be a significant issue, especially when a large number of iterations are required. For this, DiNAMIC provides *Quick Look* and *Detailed Look* platforms for the user’s options. In the first one, the original null distribution is re-used to test the significance of the most extreme markers, and thus accordingly saves a piece of computational time. In addition, the significance for multiple testing is corrected using max-T procedure exactly like STAC [4].

#### (6) GAIA [16]

In contrast to other existing methods [3], [13], [24], GAIA (Genomic Analysis of Important Alterations) incorporates within-sample homogeneity into the “peel-off” procedure under its statistical hypothesis framework: first, individual markers are randomly permuted to generate a null distribution, based on which the observed count (the number of aberrations across samples, this is equivalent to the effect of frequency of aberrations) of each marker is assessed and assigned with a significance level; second, GAIA defines a homogeneity value for each paired adjacent markers in every sample and produces a new data matrix called *H* (*N*×*M*-1), in which each element *H_ij_*∈{0, 0.5, 1}, represents maximum, medium or minimum homogeneity; finally, a homogeneous peel-off is performed on the matrix *H* to expand the boundaries of the significant regions detected previously. This “peel-off” scheme was expected to identify more recurrent CNA peaks and omit spurious peaks.

### Evaluation of the Methods

Fairly evaluating the relative merits of these methods is necessary, but this is complicated due to several realistic issues. First of all, the input data formats (segmented or raw) to different algorithms are not always the same, and those requiring segmented inputs usually adopt different segmentation algorithms. For example, the default segmentation algorithms used by STAC, GISTIC, DiNAMIC, and GAIA are GenePix Pro 4.0 [25], GLAD [22], CBS [26], and VEGA [27] respectively. Considering that different segmentation algorithms might have different abilities in processing individual CNA profiles, and thus will pose great impact on downstream analysis, we choose to use the CBS segmentation algorithm [26] for all the two-stage methods in this comparison study, since CBS is a very popular algorithm and it performs consistently well in detecting copy number changes [28]. Secondly, the significance outputs of the six methods include two types: *p*-values (STAC, KC-SMAR, CMDS, and DiNAMIC) and *q*-values (GISTIC and GAIA), and the thresholds for declaring significant in these methods are different. For a fair comparison, we choose the commonly used thresholds 0.05 for *p*-value and 0.25 for *q*-value here. Thirdly, the parameters in different methods differ greatly. For example, DiNAMIC requires an input of number of iterations, where the default setting is 10. However, such a setting is usually not large enough in real applications, since there might be a great number of aberrant markers which should be assessed. Thus, we change this default setting into a larger number in the implementation of the algorithm. For most of the algorithm parameters, we use the default settings as much as possible or the values suggested in the papers or program documents. Finally, different algorithms were written in various languages and implemented in different platforms, as shown in Table 1. This will increase the difficulties to compare the computational time of the methods in practice.

To quantitatively evaluate the performance of the methods, we test four commonly used criteria [13], [28], [29], [30] based on a large number of simulation datasets. The criteria are described in detail below.

#### 1. Type I error rate

The purpose of assessing type I error rate is to investigate the meaning of the significance levels resulted from the statistical methods for detecting recurrent CNAs [13], [30]. If type I error rate is too conservative or too aggressive, the intended meaning of the *p*-values (or *q*-values) would be reduced or lost, and it doesn’t agree with the real false positive rate in results. Thus the accuracy of the type I error rate is a critical index for evaluating methods. To this aim, we simulate a large number (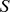) of replicated datasets with null ground truth CNAs, and calculate the type I error rate using Equation (8):
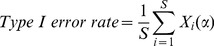
(8)where 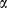 is the threshold for calling significant (e.g. 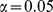), and 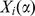 is an indicator function, i.e., if any CNAs in dataset 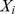 are declared significant, then 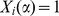; otherwise, 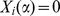. Thus, Equation (8) is actually a calculation of family-wise type I error rate [17].

#### 2. Detection power

Since CNA is a structural unit and it usually includes a number of markers, the detection power can be calculated through two ways: unit-based and marker-based calculations.

*CNA unit-based detection power:* for a ground truth (recurrent) CNA unit, it is necessary to observe how likely it can be successfully declared significant by a method. We define this detection power as the sensitivity to detect the recurrent CNA unit. Generally, exactly detecting the boundaries of (or all the markers within) the recurrent CNA unit is difficult to achieve, and this is not always necessary for locating the genes covered by CNA. For example, the genes can be mapped if a part of markers within them are overlapped by the detected CNA units. For a convenient assessment, we use the middle marker of the recurrent CNA unit to determine whether the unit is declared, i.e., if the middle marker is detected, then we suppose that the unit is successfully detected, otherwise, it is not. Accordingly, the CNA unit-based detection power of a method can be calculated by [30]

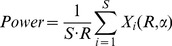
(9)where 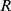 is the total number of ground truth CNA units in each simulated dataset, and 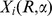 indicates the number of ground truth CNA units that are declared significant in the *i*-th dataset.

*CNA marker-based detection power:* in addition to the location of cancer driver genes, recurrent CNAs can also be used to analyze chromosomal instability index and other biological meanings [1]. So it is necessary to see how many ground truth markers are detected. Accordingly, we define this power as Equation (10) [30], in which 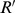 is the total number of ground truth CNA markers and 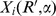 indicates the number of ground truth markers that are successfully detected in the *i*-th dataset.
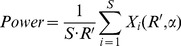
(10)


#### 3. Receiver operating characteristics (ROC) curve and AUC measure

We further assess the overall performance of the six methods, measured by both sensitivity and specificity through ROC curves, which shows how much percentage of ground truth markers are selected conditioned on a given false positive rate. Additionally, we measure the area under curve (AUC) for these methods with the purpose of evaluating their average performance especially when some ROC curves have crossed.

#### 4. Computational complexity

We evaluate the computational complexity based on execution time and memory usage. Since different methods are usually implemented in different platforms such as C++, R language, and JAVA, the comparison of the computational time might be influenced. To overcome this issue and provide a general comparison of the efficiency of the six methods, we give big-O complexities for them, in addition to the actual running times.

### Simulation Datasets

Real datasets rarely have absolutely confirmed ground truth CNAs, and thus can’t be used to evaluate the performance of the methods. However, simulation technologies provide a reasonable way to solve this problem [31]. Since the four evaluation criteria illustrated above are utilized to quantify the methods from different perspectives, it is necessary to employ different simulation schemes to generate a variety of datasets.

For the first criterion of testing type I error rate, we adopt the simulation algorithm introduced by Hsu et al [32] and Walter et al [13] to create null datasets. The algorithm is based on an instability-selection model [33], which has been originally used by many researchers to model LOH (loss of heterozygosity). The principle of simulating copy number aberrations under the instability-selection model can be simply summarized as follows [13]. The marker status is firstly denoted either by 0 as no aberration or by 1 as aberration. To generate contiguous markers which are inherent correlated along one chromosome with length of *M*, an initial marker location *x_k_* (*k*∈{1, 2, …, *M*}) is prespecified and the status of its neighboring marker *x_k_*_+1_ is then modeled based on the transition probability [13], *p_a, b_*(*d*) = *p*(*T*(*x_k_*_+1_) = *a* | *T*(*x_k_*) = *b*), where *a*, *b* = 0, 1, and *d* is the distance between adjacent markers *x_k_* and *x_k_*_+1_. Specifically, the transition probabilities have been defined as [13], [33]:
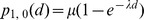


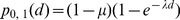
(11)where *μ* is the background or sporadic probability of aberration at a marker, and *λ* is the transition rate between regions of aberration and normality (i.e., no aberration). The other transition probabilities are *p*_0, 0_(*d*) = 1- *p*_1, 0_(*d*) and *p*_1, 1_(*d*) = 1- *p*_0, 1_(*d*). According to such probabilities, the status of the markers *x_k_*_+1_, …, *x_M_* is determined based on a Binomial distribution. For the starting marker *x_k_*, the status is assigned using a binomial random variable with probability *μ* [13]. The left part of the chromosome can be also determined similarly.

To get an idealized copy number data, the above simulation process is conducted two times, and the two simulated profiles are then combined to generate an individual sample [13]. To make the simulation data more realistic, a normal cell contamination with a random proportion ∼ Uniform (0.7, 0.9) will be added to each sample, as well as a Gaussian noise with mean 0 and standard deviation 0.25. For a more detailed description of this simulation algorithm, interested readers can refer to [33], [13], and [32].

For the second criterion of testing statistical power of the methods, we combine the features of the simulation strategies introduced by Willenbrock et al [34] and Zhang et al [9], to generate multiple ratio profiles with ground truth CNA regions, and we further consider the signal scenarios summarized by Rueda and Diaz-Uriarte such as scenarios I–III, and scenario V [14]. We create an initial data matrix in which each element is assigned with a normal copy number level. Based on this matrix, we insert the ground truth CNA regions by considering the following factors that are generally regarded to affect the statistical power of detecting recurrent CNAs: length (*L*) and amplitude (*CN*) of recurrent CNA, frequency (*F*) of recurrent CNA across samples [9], signal noise level (*σ*) of the ratio profiles, normal cell contamination (*δ*) in tumor samples [35]. To make the simulated data more realistic, we add a number of randomly placed background CNA regions to each sample. The lengths of these regions are generally similar to that of the recurrent CNAs. For the third and last evaluation criteria, we still adopt this simulation scheme but use different factor settings. Particularly for the last criterion, we focus on simulating the scale of datasets, i.e., the size of samples and the length of genome, since these are generally thought to be the main factors influencing computational complexity.

To fully investigate the performance of the six methods under different criteria, in each simulation scheme, we set the related parameters to different values to configure various data replications. The details are demonstrated in each scenario in the next.

## Results

### Performance Evaluation on Simulations

According to the four evaluation criteria and their corresponding simulation schemes, we present and analyze the comparative results of the methods and also explore and discuss the principles and features of the underlying methods.

#### Scenario 1 (Evaluation of type I error rate)

**Figure 4:**
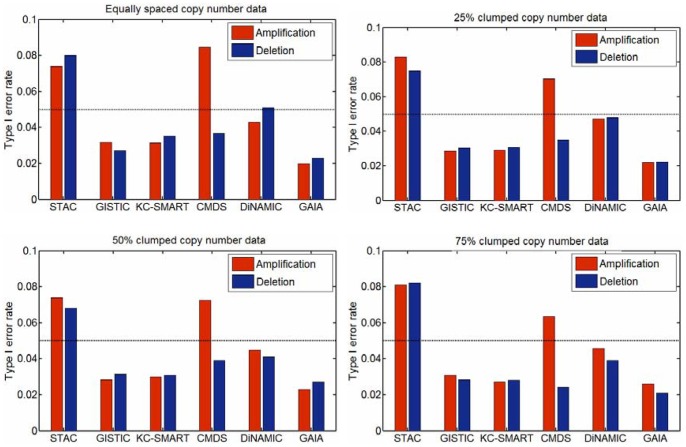
Type I error rate comparison of the six methods. Amplification and deletion are tested separately, and the corresponding results are figured with red and blue bars; the type I error rate of 0.05 is represented by the dashed line in the figure.

Based on the default parameter settings of the simulation algorithm introduced by Walter et al [13], we create four types of null datasets by altering the distance (*d*) between adjacent markers. The distance is used to emulate the density of CNA markers distributed on the genome. For example, the equally spaced copy number data means that the markers are uniformly distributed in the stretch of the genome, while 50% clumped copy number data means that half of the markers are contained in the clumps accounting for 5% of the genome size, and the other half of the markers are equally spaced in the remainder intervals accounting for 95% of the genome size. 25% and 75% clumped copy number datasets have the similar interpretations. To accurately evaluate the type I error rate for a method, under each setting of the distance *d*, we generate 10000 data replications, each of which consists of 50 samples and 2000 markers, and we calculate the type I error rates for amplification and deletion separately. The experimental results are shown in Figure 4, which suggests that the value of the type I error observed in the DiNAMIC method is closer to the significance threshold than other five methods, and the STAC method appears relatively more liberal while the GAIA method shows relatively more conservative. It should be noted that here we use the same threshold 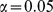 for all the methods (including *p*-value and *q*-value output methods) for a convenient comparison.

The potential explanations about the deviations from type I error rate of 0.05 are concluded based on a careful inspection of the principles behind the methods. For the STAC method, two reasons may result in a liberal type I error rate. The first reason is the partial contribution of the non-changed locations to the null distribution, since STAC creates null distribution by permuting segments obtained from individual sample analysis methods [22], [26] but not removing the non-changed segments from the genome. The second reason is that STAC tests the significance of markers on chromosome-wide or chromosome arm-wide and controls FWER only within the chromosome. From the perspective of genome-wide, the *p*-value threshold of 0.05 might be too liberal since there are usually 23 multiple tests (i.e., 23 chromosomes) in the analysis of either amplification or deletion. For the GAIA method, the primary reason for conservativeness is that it does not take into account the correlations among copy number data in both the statistic design and null distribution generation, and also it does not incorporate signal amplitude into the statistic. Theoretically, the dependency among test statistics may also lead to conservativeness [36]. In addition, extreme loci usually present higher amplitude than that of sporadic loci [1] so that the methods ignoring amplitude may decrease the detection power of extreme loci, and thus generate conservative results.

For the CMDS method, we observe that it produces a distinct difference of the type I error rates between amplification and deletion, i.e., it behaves much liberal on amplification while conservative on deletion in all the cases of Figure 4. The most likely reason is that CMDS does not deal with the overlaps of amplification and deletion in the same regions (or windows). For example, within one specific window, if a part of individuals are deleted at an average copy number *CN_d_* and another part of individuals are amplified at an average copy number *CN_a_*, the copy number mean of the window across all samples might be larger than 2, showing an amplification state, since the value of *CN_a_* (*CN_a_*∈ (2, +∞)) can be generally much larger than that of *CN_d_* (*CN_d_*∈ [0, 2)). Therefore, the deletion events tend to be masked by the amplification events from the perspective of copy number mean that has been used by CMDS to define amplification and deletion states. Accordingly, in the datasets with evenly distributed copy number gains and losses, CMDS may result in more amplification markers with low significant levels while less deletion markers.

#### Scenario 2 (Evaluation of power based on CNA units and markers)

Since amplifications and deletions of copy number are regarded to play different biological roles, and thus are usually analyzed separately for understanding cancer pathogenesis [1], [11], [18], we set out to calculate the power of the methods for detecting amplification and deletion separately. To mirror this, we first simulate three amplification events (i.e., ground truth recurrent amplifications) according to the following basic settings: *L* = {30, 20, 10}, *CN* = {3, 4, 5}, *F* = {0.08, 0.10, 0.12}, *σ* = (0.1, 0.2), and *δ* = (0.2, 0.4). *σ* = (0.1, 0.2) means a uniform distribution *U* (0.1, 0.2) from which the standard deviation *σ* is randomly drawn and a zero-mean Gaussian noise with *σ* is added to each sample. Similarly *δ* = (0.2, 0.4) indicates a uniform distribution *U* (0.2, 0.4) from which the normal cell proportion *δ* is randomly drawn for each sample. Moreover, 2 to 5 background CNA regions with lengths varying from 10 to 50 and copy numbers varying from 3 to 5 are inserted in each sample. It is worth noting that these settings have a clear interpretation: the CNA frequency across samples and its amplitude are inversely proportional to the CNA length [11].

**Figure 5:**
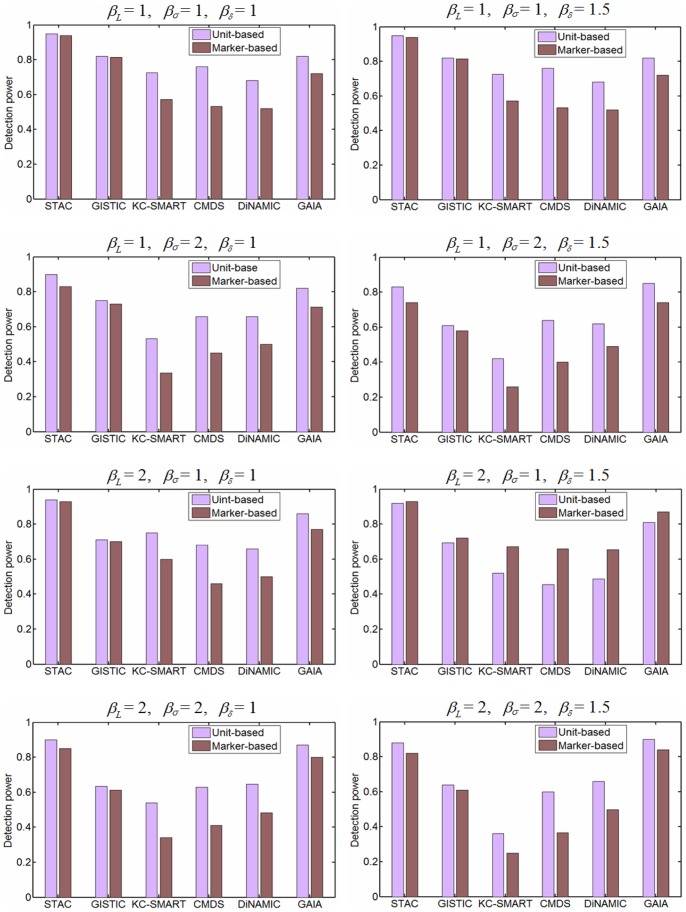
Power comparison of the six methods. Unit-based and marker-based powers are tested on CNA amplifications, and the values for each parameter are calculated based on 50 simulated replications, which are depicted with pink and dark red bars respectively.

To generate various datasets for understanding how the factors (*L*, *σ*, *δ*, et al.) influence the power of the methods, we use three parameters *β_L_*, *β_σ_*, and *β_δ_* to modify the lengths of the recurrent CNAs, the noise level, and the normal cell contamination of the copy number profiles. Under each configuration, we simulate 50 replications with log_2_-ratio values, each of which contains 50 samples and 2000 markers. The unit-based and marker-based detection powers are calculated using Equations (9) and (10), respectively, and the corresponding results are shown in Figure 5. Similar to the analysis of amplification, we also evaluate the detection power for the methods by simulating deletion events (i.e., ground truth recurrent deletions). The results are presented in Figure S1.

**Figure 6:**
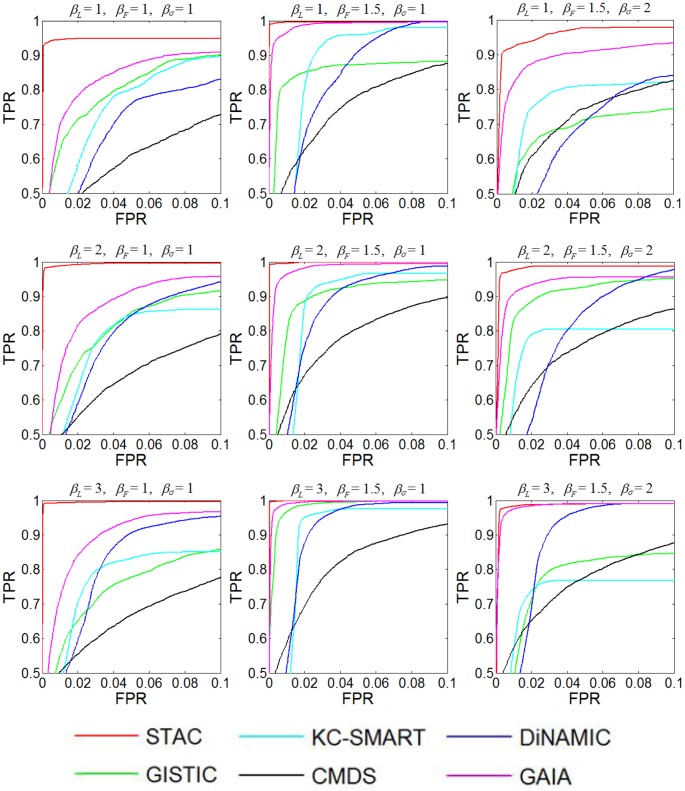
ROC curve comparison of the six methods. Nine parameter settings are considered for comparing true positive rate (TPR) vs false positive rate (FPR) of the methods when testing CNA amplifications. The values of TPR and FPR in each parameter are averaged over 50 simulated replications.

As shown in these figures, most methods achieve slightly higher power in the case of lower levels of noise and lower fractions of normal cell contamination, and the unit-based power is usually higher than the marker-based power, implying the generally recognized fact that the boundaries of recurrent CNA regions are more difficult to detect than the middle loci of the regions [16], [28], [37]. Moreover, it can be noted in the figures that the STAC method is more sensitive to recurrent CNAs in most cases than other methods. Examination of the principles underlying STAC reveals two reasons for this advantage. One contributor is the “footprint” statistic, which accounts for the lengths of aberrant intervals covering the markers being tested and directly measures tight alignment of the intervals (the intervals are contained in different samples and are defined as a stack) [4]. The tight alignment is a reasonable reflection of the concordance of CNAs. The second and more important reason is that STAC generates the null distribution corresponding to the number of intervals in a stack covering the tested marker. For example, if one stack contains 10 intervals (i.e., there are 10 samples aberrant in the same region), in order to assess the significance of the anchor markers contained in the stack, STAC calculates the footprints of all stacks that consists of exactly 10 intervals on permuted data, and then compares the observed statistic values to these footprints to get *p*-values. Theoretically, such null distributions account for subspace of the whole samples and thus facilitate finding consistent CNAs within subsets of samples. Therefore, the combination of the “footprint” statistic and the null distribution reduces the influence of highly frequent copy number aberrations across whole samples on detecting less-extreme markers.

**Table 2:** The area under curve (AUC) measurements of the six methods under various simulation configurations.

	STAC	GISTIC	KC-SMART	CMDS	DiNAMIC	GAIA
*β_L_* = 1, *β_F_* = 1, *β_σ_* = 1	0.976	0.907	0.924	0.899	0.863	0.956
*β_L_* = 1, *β_F_* = 1.5, *β_σ_* = 1	0.998	0.933	0.973	0.927	0.962	0.996
*β_L_* = 1, *β_F_* = 1.5, *β_σ_* = 2	0.961	0.866	0.894	0.929	0.907	0.962
*β_L_* = 2, *β_F_* = 1, *β_σ_* = 1	0.995	0.947	0.918	0.929	0.880	0.969
*β_L_* = 2, *β_F_* = 1.5, *β_σ_* = 1	0.999	0.948	0.977	0.961	0.976	0.998
*β_L_* = 2, *β_F_* = 1.5, *β_σ_* = 2	0.984	0.887	0.873	0.939	0.917	0.978
*β_L_* = 3, *β_F_* = 1, *β_σ_* = 1	0.998	0.921	0.943	0.951	0.930	0.987
*β_L_* = 3, *β_F_* = 1.5, *β_σ_* = 1	0.999	0.985	0.969	0.961	0.983	0.998
*β_L_* = 3, *β_F_* = 1.5, *β_σ_* = 2	0.997	0.886	0.893	0.950	0.959	0.995

**Figure 7:**
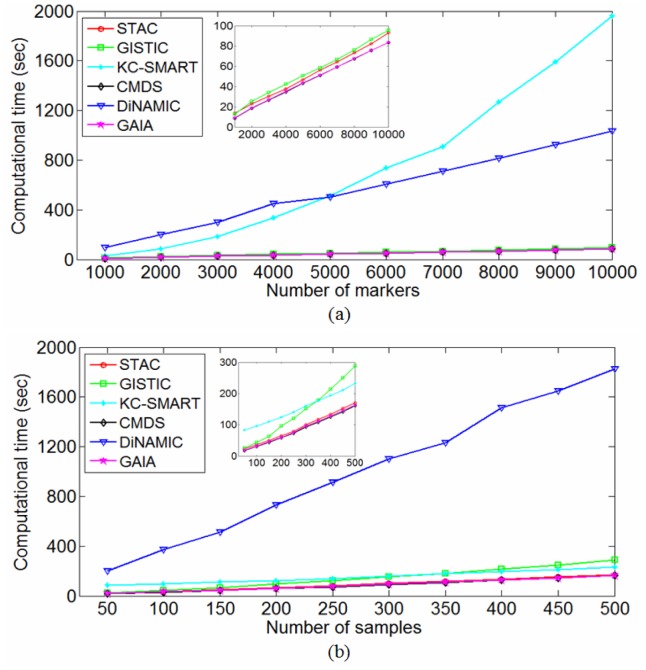
Comparison of execution times for the six methods. Computational time is depicted as a function of genome size when setting the sample size to 50, and is then depicted as a function of sample size when setting the genome size to 2000.

#### Scenario 3 (ROC curve and AUC comparison of the six methods)

Since the identification of recurrent CNAs usually incorporates more or less false positives in the results, it is necessary to see how many ground truth CNAs can be identified given a false positive rate. For this purpose, we employ the similar simulations of scenario 2 to evaluate sensitivity-specificity ROC curves for the six methods. The setting of the normal cell proportion *δ* is now changed to: *δ* = (0.2, 0.8), and we further use an additional parameter *β_F_* to alter the frequency of recurrent CNAs across samples, aiming to increase the variety of simulations. Again, in each factor configuration, we simulate 50 replicated log_2_-ratio datasets each with 50 samples and 2000 markers. The sensitivity (true positive rate, TPR) and specificity (false positive rate, FPR) are calculated for each method by ordering the markers according to their significance values, and then are averaged over the 50 replications. The results obtained from the analysis of amplifications are demonstrated in Figure 6, from which we clearly see that the STAC and GAIA methods perform consistently well. It is important to note that in Figure 6 we focus on the top-left part of the ROC curves. Specifically, we start TPR from 0.5 in Y-axis and end FPR at 0.1 in X-axis, since such curves would provide a clearer observation of the difference between the methods than those ranging from 0 to 1 in both X and Y axis. Moreover, a TPR level higher than 0.5 and a FPR level lower than 0.1 are usually required in real problems especially in the analysis of high-resolution datasets, due to the need of controlling true and false discovery rates [9].

**Figure 8:**
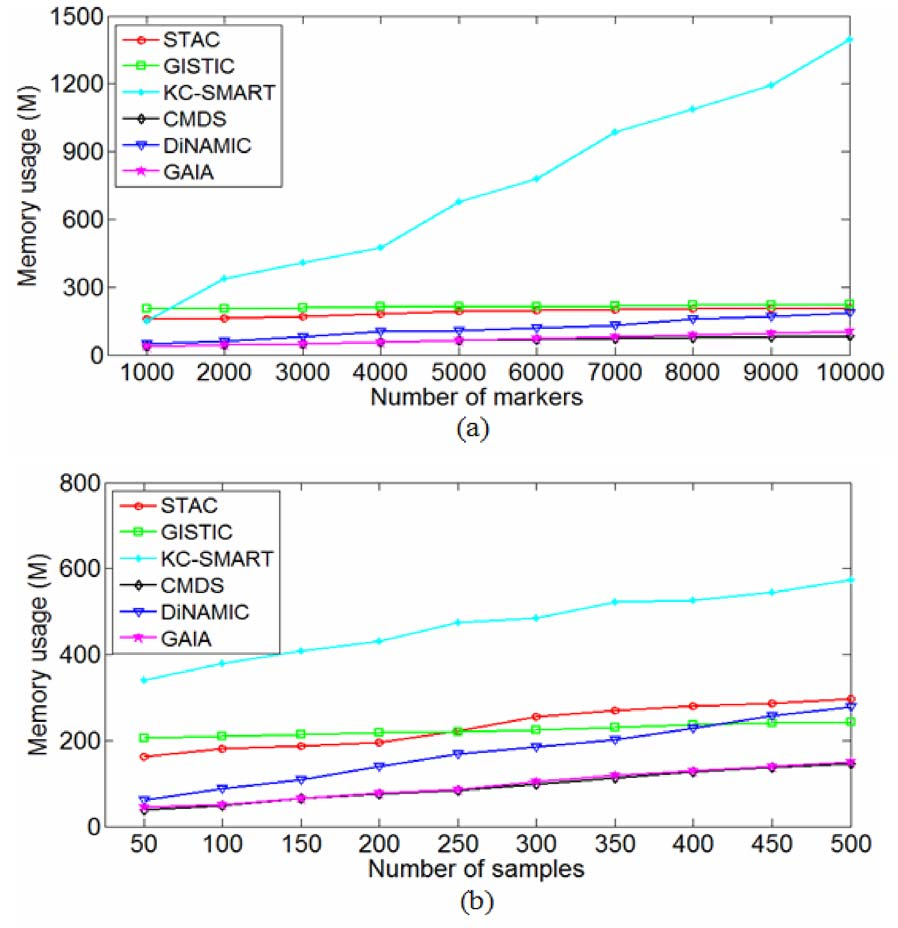
Comparison of memory usages for the six methods. Memory usage is depicted as a function of genome size when setting the sample size to 50, and is then depicted as a function of sample size when setting the genome size to 2000.

Under the same simulation configurations, we measure the area under curve (AUC) for the six methods. The results are shown in Table 2, from which one can find that STAC and GAIA have the highest AUC values in most cases. The observation from this comparison is roughly consistent with that from the ROC curve comparison.

Additionally, to test the methods in analyzing deletions of copy number, we make a comparison of ROC curves in Figure S2, where we can also see the advantages of STAC and GAIA.

**Table 3:** Comparison of big-O computation complexity for the six methods.

Method	Time complexity	Memory complexity
STAC	O((*T*+1)*NL*)	O(*NL*)
GISTIC	O(*NL*)	O(*NL/l*)
KC-SMART	O(*TNL*log*L*)	O(*TNL*+*NL*log*L*)
CMDS	O(*NL*)	O(*NL*)
DiNAMIC	O(*TNL*+*L*)	O(*NL*)
GAIA	O(*NL*)	O(*NL/l*)

#### Scenario 4 (Assessment of computational complexity)

Computational complexity of a method is a key factor for its application to real data due to the huge size of the genome being analyzed. Generally, three factors are regarded to determine the computational complexity, sample size, genome length, and algorithm for analyzing the data. To achieve a reasonable comparison of computational complexities for the six methods, we perform experiments using modest computational resources, available to all researchers. Specifically, our computer platform is: Linux OS (Ubuntu 9.04) and Windows XP OS, 5.99 GB RAM, and 2.00 GHz CPU. Various simulation datasets are generated by changing genome length or sample size based on the simulation scheme illustrated in previous text. Specifically, we set the sample size to 50 and change the genome length from 1000 to 10000, and then set the genome length to 2000 and change the sample size from 50 to 500 in the data simulations, with the purpose of observing the changes of running time with respect to the genome length and sample size. In each setting of parameters (i.e., sample size and genome length), we simulate 50 replicated datasets, and measure the running times and memory usages of the six methods by averaging over the replications. In addition, for the two-stage methods, including STAC, GISTIC, DiNAMIC, and GAIA, we add the costs of the preprocessing of datasets (i.e., single-sample analysis) to the running times for making a fair comparison. The comparative results of the running times are depicted in Figure 7, which suggests that CMDS and GAIA are the fastest, while KC-SMART and DiNAMIC are the slowest. It is worthy noting that in Figure 7 the efficiency of KC-SMART is seriously affected with the increased genome length while it is slightly affected with the increased sample size. Similarly, we present the comparative results of the memory usages in Figure 8, which suggests that CMDS and GAIA require the lowest memory size while KC-SMART requires the highest memory size. Here, it is important to note that the results shown in Figures 7 and 8 should not be strongly platform-dependent since the machine memory is sufficient for the six algorithms to analyze the simulation datasets that we have considered.

**Figure 9:**
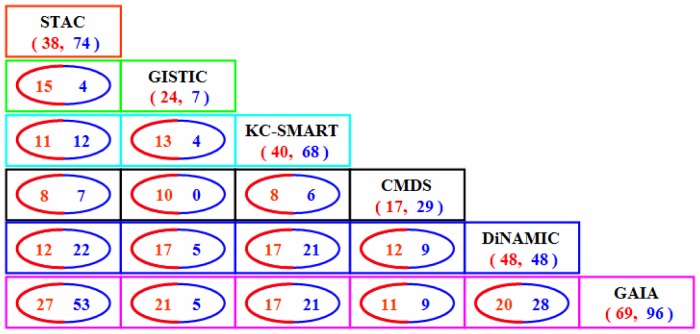
Result comparison of the six methods on the lung adenocarcinoma dataset. The red and blue colored numbers indicate the numbers of amplified and deleted recurrent CNA regions, respectively. Specifically, (*N_A_*, *N_D_*) (e.g., (38, 74)) in each box means that there are *N_A_* amplified regions and *N_D_* deleted regions identified by the corresponding method; and the cycled numbers denote the numbers of overlapped regions detected by the corresponding two methods. For example, in the location of the first column and the last line in the figure, ‘27′ represents the number of amplification regions overlapped between STAC and GAIA, while ‘53′ represents the number of overlapped deletion regions.

**Table 4:** Comparison of the number of lung cancer related regions that are identified by the six methods on the lung adenocarcinoma dataset.

	STAC	GISTIC	KC-SMART	CMDS	DiNAMIC	GAIA
# of lung cancer related amplifications	16	17	13	10	18	22
# of lung cancer related deletions	17	5	9	8	12	21

Different algorithms were written with different languages such as R, Matlab, and Java, thus it is insufficient to assess the computational complexities of the method only using the practical running times and memory usages. To provide a general comparison of the methods, we examine the principles of the algorithms carefully and give big-O time costs and memory requirements for them in Table 3, where *N* denotes the sample size, *L* denotes the genome length (number of markers), *T* represents the number of permutations, and *l* is the averaged length of the segments obtained from the single-sample analysis of the whole genome. Comparing Figure 7, Figure 8, and Table 3, we find that the big-O computational complexities are roughly consistent with the running time and memory usage results.

**Figure 10:**
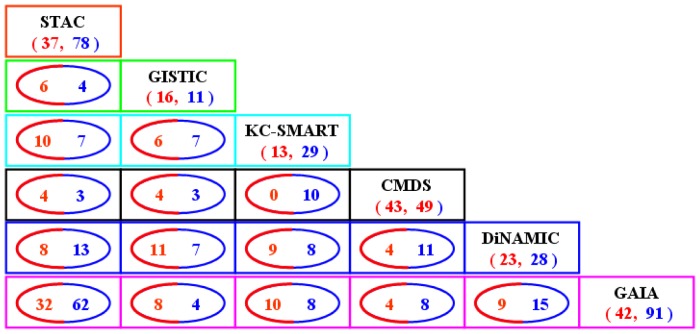
Result comparison of the six methods on the glioblastoma dataset. The red and blue colored numbers indicate the numbers of amplified and deleted recurrent CNA regions, respectively. Specifically, (*N_A_*, *N_D_*) (e.g., (37, 78)) in each box means that there are *N_A_* amplified regions and *N_D_* deleted regions identified by the corresponding method; and the cycled numbers denote the numbers of overlapped regions detected by the corresponding two methods. For example, in the location of the first column and the last line in the figure, ‘32′ represents the number of amplification regions overlapped between STAC and GAIA, while ‘62′ represents the number of overlapped deletion regions.

### Real Applications

To investigate the behaviors of the methods in real applications, we first test them on a cancer dataset with 371 lung adenocarcinoma samples [2], [9], [17]. Raw CNA profiles are segmented using the CBS algorithm [26] and are transformed into the input formats to the two-stage methods. Since this dataset has been already analyzed by the GISTIC and CMDS methods in their papers [3], [9], we do not run them again but only use their presented results for comparing to other four methods (STAC, KC-SMART, DiNAMIC, and GAIA). Recurrent copy number alterations usually include both arm-level and focal events, which are regarded to have different lengths and distinct biological consequences [3], [11]. Specifically, focal events are generally defined as the peak regions with the most significance levels among the recurrent regions. Here, we focus on the comparison of focal events being identified by the six methods, since these events encompass limited number of genes and usually contain cancer driver genes [2], [11]. To mirror this, we employ the log_2_-ratio thresholds of 0.848 and −0.737 for defining amplifications and deletions to fit those methods (STAC, GISTIC, and GAIA) that require copy number calls. Actually, these thresholds are the default settings of GISTIC in its applications [2], [3].

**Table 5:** Comparison of the number of glioblastoma related regions that are identified by the six methods on the glioblastoma dataset.

	STAC	GISTIC	KC-SMART	CMDS	DiNAMIC	GAIA
# of glioblastoma related amplifications	10	11	7	6	10	14
# of glioblastoma related deletions	10	4	7	4	5	12

We produce the result of each method using its default significance thresholds as aforementioned. For example, in the STAC method, we use a *p*-value of 0.05 as a cutoff for declaring significant, and in the GAIA method, we use a *q*-value of 0.25 as the cutoff. The results derived by the methods are presented in Table S1. Since there are no definite ground-truth driver genes in real datasets, it is extremely difficult to quantify the performance of the methods using the previously described criteria (e.g., power and true positive rate vs false positive rate). Instead, we first compare the total number of identified recurrent regions of the six methods, and give the numbers of overlapped regions between any two methods (shown in Figure 9). This will help observe which method is the highest concordant one, i.e., which method overlaps other methods in the largest area. From Figure 9, we observe that GAIA overlaps the largest area with other methods. Specifically, GAIA overlaps STAC with 80 regions and averagely overlaps 42.4 regions with the other five methods. From the perspective of overlapping percentage, up to 83.9% of the regions in GISTIC and 71.4% of the regions in STAC are overlapped by GAIA. We also find (Table S1) that there are seven regions including 1q21.2, 7p11.2, 8q24.21, 11q13.3, 12q15, 14q13.3, and 17q12 are overlapped by all the six methods. Most of these regions have been validated to encompass or closely near to cancer related genes such as MDM2, MYC, EGFR, ERBB2, CCND1, ARNT [2], [9].

Moreover, based on a collection of previous studies on lung cancers and their subtypes [2], [38], [39], [40], [41], [42], [43], [44], [45], [46], [47], [48], [49], [50], [51], [52], [53], [54], [55], [56], we compare the methods using the numbers of lung cancer related regions that are identified by them. The comparative result is presented in Table 4. Here it should be noted that we have only counted the regions reported to be associated with lung cancer in previous studies, the remainder regions in the result of each method are generally regarded as candidates and need further investigation in terms of their biological relevance.

Similar to the analysis of lung adenocarcinoma dataset, we further compare the methods based on a set of glioblastoma samples [3], [57]. We still use the log_2_-ratio thresholds of 0.848 and −0.737 for defining amplification and deletion markers, and also use the result already reported by the GISTIC method [3] in this comparison. The numbers of recurrent regions identified by the six methods and the numbers of overlapped events among them are presented in Figure 10, from which we see that GAIA detects the largest number of regions including 42 amplifications and 91 deletions, and also overlaps the largest area with other five methods. For example, GAIA overlaps STAC with 94 (81.7% of 115 regions identified by STAC) events and overlaps DiNAMIC with 24 (47% of 51 regions identified by DiNAMIC) events. The detailed information of the identified regions by the six methods is given in Table S2. Based on a large collection of previous studies on glioblastoma patients [3], [58], [59], [60], [61], [62], [63], [64], [65], [66], [67], [68], [69], [70], [71], [72], [73], [74], [75], we summarize the numbers of glioblastoma related regions resulted by the methods for a further comparison. This is shown in Table 5.

It should be noted that in this comparison we have used the default segmented profiles [3] of the glioblasotma samples for both the one-stage and two-stage methods. Specifically, we test the KC-SMART and CMDS methods on the segmented data rather than raw data, since these two methods also allow for segmented input signals [8], [9]. This will help to investigate the performance of the methods on different data platforms. Comparing to the result of the lung adenocarcinoma dataset (Figure 9), most of the methods identify less regions in the glioblastoma dataset (Figure 10). However, the number (92) of regions detected by CMDS in Figure 10 is significantly larger than that (46) in the lung adenocarcinoma dataset. The most likely reason is that the noise level of the segmented profiles is significantly lower than that of raw profiles, and the underlying principle of CMDS is to distinguish recurrent copy number alterations from noise copy number. Therefore, the input signals to CMDS with lower noise level would result in more regions.

## Discussion

### Brief Summary

Six representative methods for identifying recurrent copy number alterations are examined and compared in this paper. It is not straightforward to state which method is the best one. However, according to experimental results on a larger number of simulation datasets, we find that the STAC and GAIA methods perform consistently well except for their liberal and conservative significance levels. By observing the results under the three performance assessment criteria (i.e., type I error, power, and ROC), we further find that the behavior of type I error does not has a clear relationship with that of power and ROC, while the behavior of power displays a linear relationship with that of ROC curves. In terms of computational complexity, KC-SMART is heavily affected by the increased genome size while slightly by the sample size. This is mainly because KC-SMART assigns *KSE* score to each marker by incorporating information of a large number of neighboring markers, which needs much computational time. DiNAMIC is also the slowest method, which provides two options (*Quick Look* and *Detailed Look*) for statistical significance assessment. It should be noted that in our experimental comparison, we always choose the *Quick Look* scheme in DiNAMIC, which needs relatively less computational time when compared with *Detailed Look* scheme with time complexity of O(*T′TNL*), where *T′* denotes the number of iterations. If we test all the markers in the genome, *T′* equals to *L*. The methods requiring implementation of permutations are generally much slower than those without permutations.

In the applications on the lung adenocarcinoma and glioblastoma datasets, the six methods identify different numbers of recurrent CNA regions and display various pair-wise overlaps (Figure 9 and Figure 10). Among these methods, GAIA identifies the largest number of recurrent regions and shows the highest concordance with other methods, and it further provides the largest number of lung cancer and glioblastoma related regions (Table 4 and Table 5). Nevertheless, the proportion of the cancer related regions to the identified regions in GAIA is not as high as that in GISTIC (e.g., 70.8% of 31 regions resulted from lung cancer dataset). In addition, the STAC method also shows a high concordance performance either in method comparison (Figure 9 and Figure 10) or cancer related gene comparison (Table 4 and Table 5). Inevitably, the values in Table 4 and Table 5 might contain some bias, since a large number of new studies on lung cancer and glioblastoma are emerging, making it difficult to track every novel discovery in this area especially for those being published or unpublished. In these real applications, another observation is that most methods identify more deletion regions than amplification regions, but reveal relatively less deletion regions associated with lung cancer or glioblastoma. The most common explanation is that the homogeneous deletions are very frequent in the lung cancer profiles, and the threshold (−0.737) for defining deletions is critical so that most homogeneous deletions are preserved but some heterogeneous deletion regions (low-to moderate-amplitude) with biological relevance might be filtered out [11].

### Challenging Issues

Although a number of methods have been developed for the identification of recurrent copy number alterations in multiple samples and new methods with more sophisticated capabilities continue to emerge, several important and challenging issues in this area have not yet been well solved. In the first place, the relationship between significant, recurrent, and cancer driver CNAs is less modeled in a decent way. Many existing algorithms stop after obtaining significant CNA regions. Practically, the significant regions may contain a huge number of genes, and not all of them are the cancer driver genes. Accordingly, only identifying significant regions is not enough to define biological events, since the number of cancer driver genes is generally limited. Thus, distinguishing driver mutations from significant or recurrent CNAs is a critical and challenging issue. Interestingly, GISTIC performs a further step to define “peak regions” with the most significant levels from each significant region and employs a relatively high threshold (e.g. over 3.6 copies of amplifications and less 1.2 copies for deletions) to detect focal regions, which contain limited numbers of genes [3]. Considering that the high threshold may filter out low-to moderate-amplitude focal events, a new version called GISTIC2.0 was proposed to separate the whole genome into arm-level and focal regions, which were then analyzed respectively by estimating the corresponding background rates [11]. The focal events will be paid much attention to locate biologically relevant genes.

The second challenge is to detect driver aberrations for cancer subtypes, since most cancers are heterogeneous [76], [77] and each subtype displays distinct copy number patterns [1], [4]. The answer to this question will help much in providing important information for diagnosis and treatment of subtypes. Many existing methods have already tried to investigate the CNA patterns in cancer subtypes. For example, STAC utilizes unsupervised two-way hierarchical scheme to cluster samples based on its detected regions [4], and pREC-S identifies subsets of samples that share regions of CNA based on Hidden Markov Model (HMM) [14], [78]. Some other methods use clinical information (e.g., primary vs recurrent, high grade vs low grade, and survival time) or phenotype to divide cancer samples into subsets and then analyze each subset separately [1], [79]. However, many cancers may be involved with highly complicated heterogeneous structures. For example, in the high grade ovarian cancer subtype, different samples have different survival times and distinct copy number patterns. This greatly increases the difficulties of defining small cancer subtypes and identifying characteristic CNA regions associated with specific subtypes.

### Potential Extensions

Based on previous analysis and discussions, we conclude several potential directions that can be pursued to improve the identification of driver copy number alterations. First, the advantages of the existing methods can be combined to refine the methodology. For instance, STAC can be refined by incorporating the peel-off scheme of the GISTIC method, so as to detect a set of “peak regions” from the significant STAC regions. This would be beneficial for reducing the scale of the identified regions and improving the purity of cancer driver alterations in the result. Second, one issue that has not been taken into consideration in the current methods is the contribution of the null markers (i.e., not changed markers) to null distributions. Specifically, when the threshold for defining aberrations is very high, a great number of markers are filtered out as null markers, which are not removed from the genome when performing permutations and generating the null distribution. Consequently, the mean of the null distribution is theoretically a little biased to the left, from the viewpoint of distinguishing recurrent CNAs from random background aberrations. Although it is not clear how much benefit could be obtained by removing the contribution of the null markers, this can be worthy of trying. Third, since the samples within a cancer subtype may themselves be heterogeneous (e.g., high grade ovarian cancer), only performing one-dimensional (i.e., across-genome) permutations in the whole samples may achieve a limited number of subtype-specific CNA events. The suggestion here is to adopt two-dimensional (i.e., across genome and sample spaces) permutations to establish the null distribution. One possible way to realize this is to incorporate the principle of pREC-S [78] and bootstrap sampling scheme into one of the six methods. Finally, analyzing absolute copy numbers in somatic DNA alterations of cancers would be much helpful [17], [80], [81], in either identifying cancer driver genes or exploring intra-tumor heterogeneity. Currently, most existing methods have only handled the relative copy number data that are generated directly from array or sequencing experiments.

## Supporting Information

Figure S1 Power comparison of the six methods by testing CNA deletions. The unit-based and marker-based powers for each parameter are calculated based on 50 simulated replications, which are depicted with pink and dark red bars respectively.
(DOC)

Figure S2 ROC curve comparison of the six methods by testing CNA deletions. Nine parameter settings are considered for comparing true positive rate (TPR) vs false positive rate (FPR) of the methods. The values of TPR and FPR in each parameter are averaged over 50 simulated replications.
(DOC)

Table S1 **Recurrent CNA regions identified by the methods for the lung adenocarcinoma dataset.**
(XLS)

Table S2 **Recurrent CNA regions identified by the methods for the glioblastoma dataset.**
(XLS)
