# Supplementary material for: Comparative Analysis of Methods for Identifying Recurrent Copy Number Alterations in Cancer
Source: PLoS One. 2012 Dec 20;7(12):e52516. doi: 10.1371/journal.pone.0052516 (PMC3527554; doi:10.1371/journal.pone.0052516)
Supplement: Figure S1 — Power comparison of the six methods by testing CNA deletions. The unit-based and marker-based powers for each parameter are calculated based on 50 simulated replications, which are depicted with pink and dark red bars respectively. (DOC) [file pone.0052516.s001.doc]

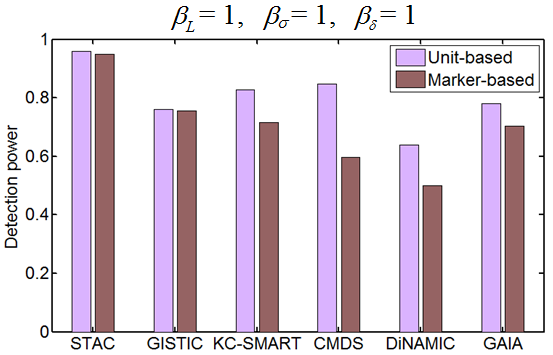

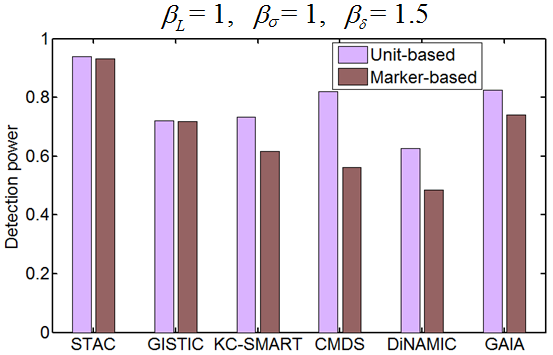


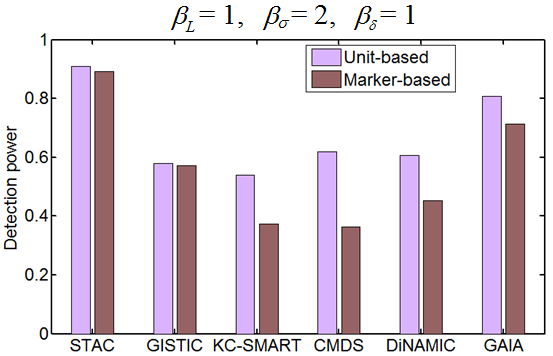

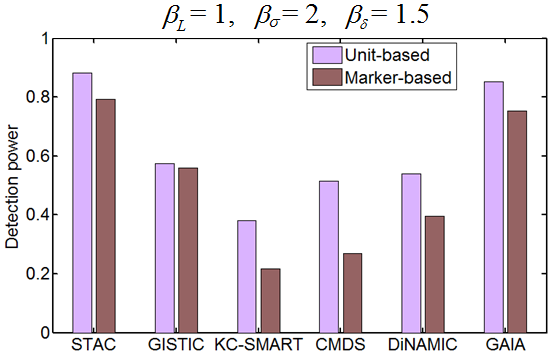


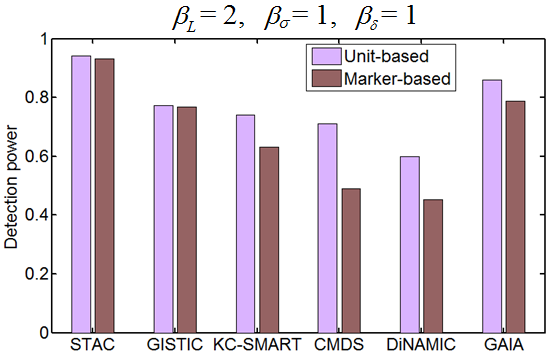

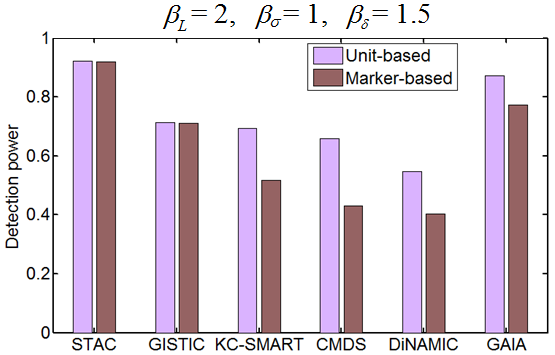


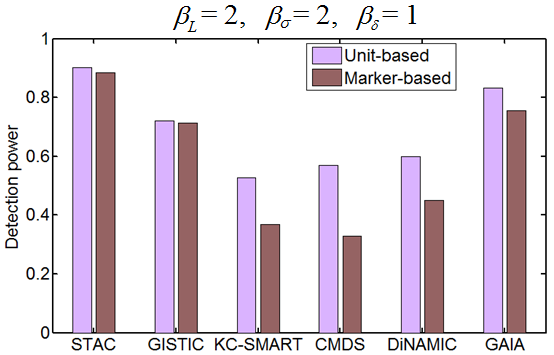

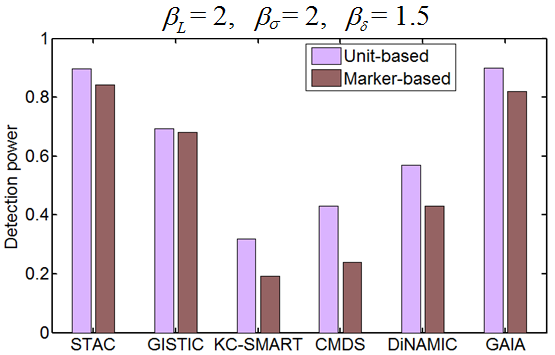


**Supplementary Figure 1.** Power comparison between the six methods (STAC, GISTIC, KC-SMART, CMDS, DiNAMIC, and GAIA) in identifying deletion events. The result in each parameter configuration is based on 50 replicated simulation datasets.
