# Supplementary material for: Comparative Analysis of Methods for Identifying Recurrent Copy Number Alterations in Cancer
Source: PLoS One. 2012 Dec 20;7(12):e52516. doi: 10.1371/journal.pone.0052516 (PMC3527554; doi:10.1371/journal.pone.0052516)
Supplement: Figure S2 — ROC curve comparison of the six methods by testing CNA deletions. Nine parameter settings are considered for comparing true positive rate (TPR) vs false positive rate (FPR) of the methods. The values of TPR and FPR in each parameter are averaged over 50 simulated replications. (DOC) [file pone.0052516.s002.doc]

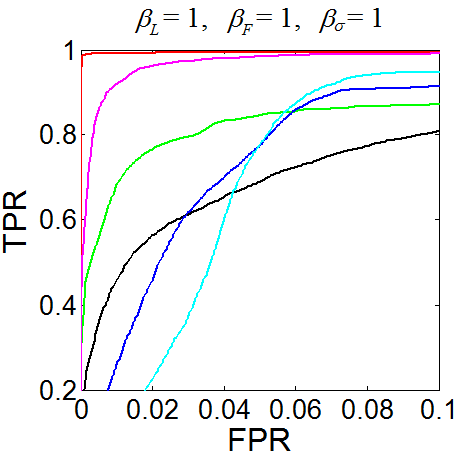

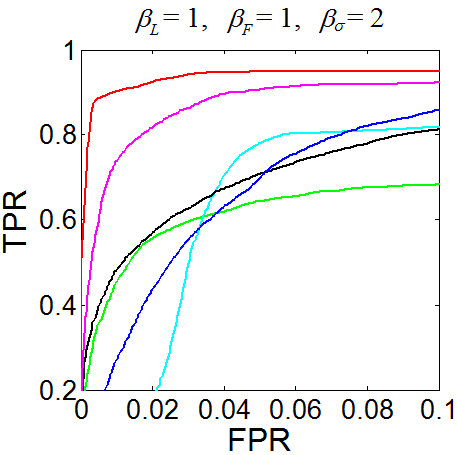

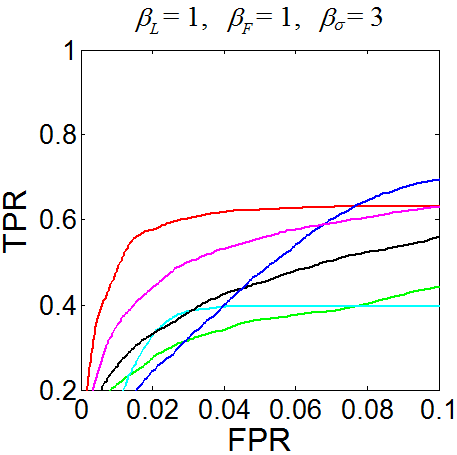

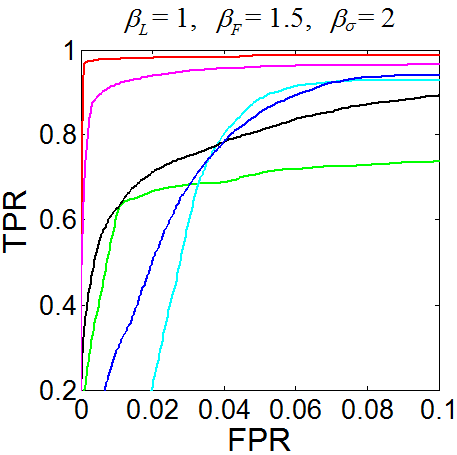

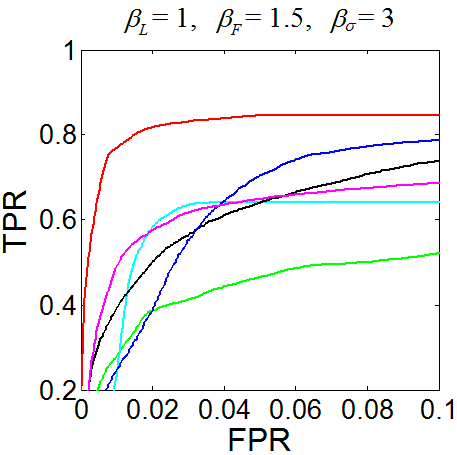

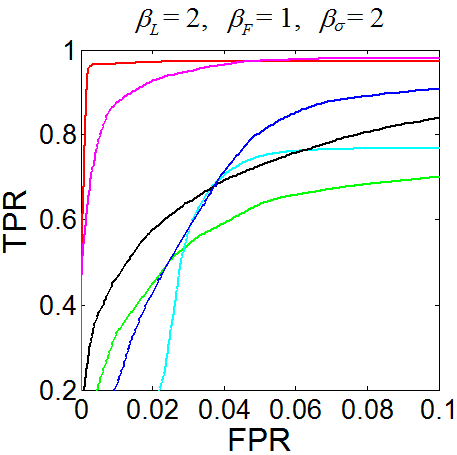

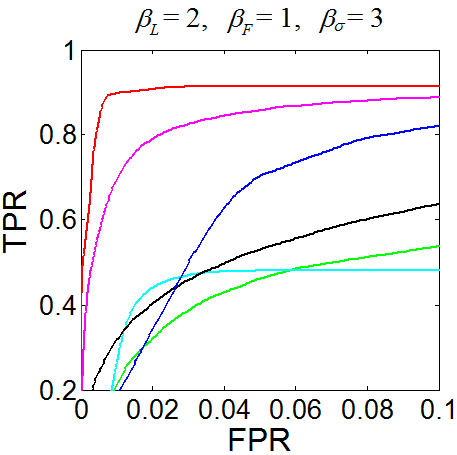

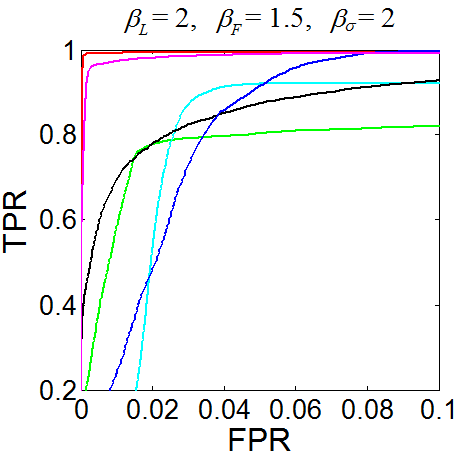

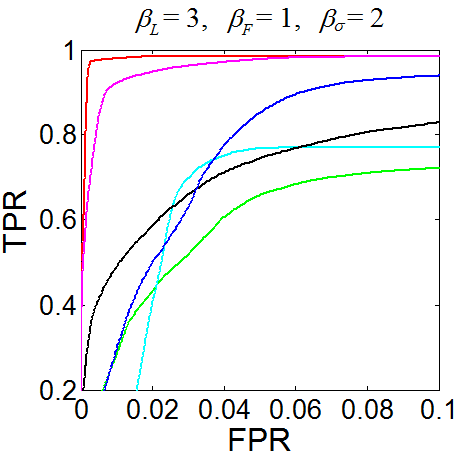

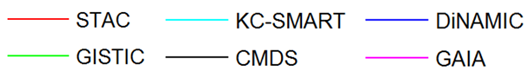


**Supplementary Figure 2.** Performance evaluation of six methods (STAC, GISTIC, KC-SMART, CMDS, DiNAMIC, and GAIA) on realistic simulation of deletion copy number data sets, quantified by the partial ROC curves (north-west) (TPR: true positive rate; FPR: false positive rate). The results are the averages calculated based on 50 replications under each of various parameter settings.
